# Management of Deep Orbital Dermoid Cysts

**DOI:** 10.4103/0974-9233.53376

**Published:** 2008

**Authors:** Imtiaz A. Chaudhry

A 34-year-old man was referred for evaluation of his gradual proptosis of left eye for many years. He had no prior history of trauma or eye surgery and his family history was non-contributory. On examination his un-corrected visual acuity was 20/20 in the right eye and 20/60 in the left eye. His intraocular pressures were normal in both eyes. He had 6 mm of left-sided proptosis, limitation of supra-duction and abduction.(Fig 1A) He had no relative afferent pupillary defect and fundus examination revealed retinal strea on the left side. Computed tomography scan revealed a large cystic lesion occupying most of the supra-temporal aspect of left orbit reaching up-to the apex resulting in proptosis and pushing the optic nerve medially. In addition, there was bony erosions of the left orbital roof, lateral wall and the floor. (Figs 1B and 1C) Diagnosis of deep orbital dermoid was made based on clinical history and imaging studies. A lateral orbitotomy was made through the extended upper eyelid crease incision. (Fig 1D) The wall of the large cystic lesion was approached and cystic lesion aspirated with 25-gauge needle attached to a syringe resulting in its collapse. (Fig 1E) Blunt dissection around the lesion dislodged it from apex of the left orbit. (Fig 1F) The area was irrigated with corticosteroids solution and wound was closed after a drain was placed. Post-operatively, patient had improvement in his left eye visual acuity, proptosis and motility. (Fig 1G)

**Figure 1:**
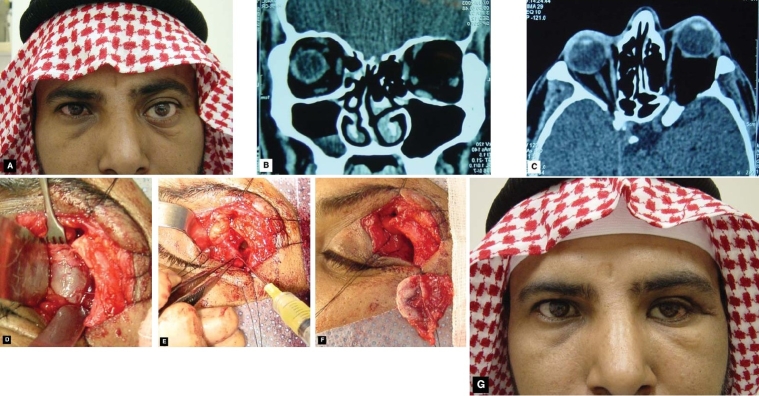
External photograph of a 34-year-old male who presented with a gradually progressive painless left proptosis.(A) Computed tomography scan revealed left eye proptosis and cystic lesion behind left eye globe pushing the optic nerve medially and expansion of left orbit and erosion of the roof, floor and lateral wall, (coronal B; axial C). Exposure of the deep dermoid cyst through lateral orbitotomy incision (D), controlled aspiration of the cystic fluid (E), and complete removal of the wall of the cyst from the apex of left orbit. (F) Two-week post-operative photograph of the same patient showing slight enophthalmos on the left side. (G)

## Comments

Dermoid cysts are among the most common congenital orbital lesions. These cysts arise from nests of embryonic epithelium left between fetal lines of closure. In one of the reported histopathologic survey of 307 orbital tumors, 35% were found to be dermoid cysts.^1^ In Shield's survey of 645 orbital biopsies of all age groups, 24% were dermoid cysts; however, among the 250 children under 18 years, 46% were dermoid cysts.^2^,^3^ Dermoid cysts were also found to be the most common orbital tumors among the 174 histopathologically proven lesions in a study by Iliff and Green.^4^ In their survey, more than 70% of orbital dermoid cysts were diagnosed before the age of 5 years.

Dermoid cysts are classified according to the site of their attachment in relation to the orbital rim either exophytic or endophytic. Exophytic dermoid cysts grow externally and are discovered early in childhood, while endophytic cysts like the patient described in this essay grow internally and are discovered later in youth or during adulthood years. Shields described dermoid cyst location as either juxta-sutural, sutural or free with no contact with intra or extra orbital bones.^2^,^5^ Juxta-sutural dermoid cysts are located adjacent to bony sutures and the dermoid cyst extends into bony sutures. Zygomatico-frontal dermoid cysts are located superotemporally and account for the majority of the cysts encountered. Maxillofacial dermoid cysts are located at the supra-nasal area and is the second most common location for these lesions. Imaging studies such as ultrasonography, computed tomography and magnetic resonance may help delineate the extent, depth and relationship of deep dermoid with surrounding tissues and eye globe.

Indications for treatment of orbital dermoid cysts are cosmetic, recurrent inflammation or risks of amblyopia. While there is minimal risk of amblyopia, majority of the dermoid cyst are removed because of family's concern for growing lesions.^5^,^6^,^7^ Also, episodes of recurrent inflammation after direct trauma necessitate excision of these cysts. Types of incisions could be direct over the dermoid cyst through the sub brow or over the eyelid crease. Dissection is carried around the lesion without rupturing the cyst. However, when the cyst is large, deep in orbit and filled with fluid as in this patient, it may be wise to aspirate with 25 gauge needle to decompress the lesion first. This approach can help to keep the external incision small and also aid in gentle dissection around the lesion deep into the orbital cavity. The dermoid cyst should be completely removed from its attachments to the bony sutural lines. The area should be thoroughly irrigated with corticosteroids solution especially if there is any spillage of cystic contents. The incision is usually closed in 2 layers; deep layer should be closed with 6.0 chromic and superficial layer with 6.0 plain-gut especially in children, as suture removal is difficult in this age group.
